# Hashimoto’s encephalitis associated with AMPAR2 antibodies: a case report

**DOI:** 10.1186/s12883-017-0823-4

**Published:** 2017-02-21

**Authors:** Mingqin Zhu, Xuefan Yu, Caiyun Liu, Chenchen Duan, Chunxiao Li, Jie Zhu, Ying Zhang

**Affiliations:** 1 Department of Neurology and Neuroscience Center, First Hospital of Jilin University, Xinmin Street No 71, Changchun, 130000 China; 2 Division of Neurodegerneration, Department of Neurobiology, Care Sciences & Society, Karolinska Institutet, SE 14157 Huddinge Stockholm, Sweden

**Keywords:** Limbic encephalitis, Autoimmune thyroid disease, AMPAR, Hashimoto’s thyroiditis, Hashimoto’s encephalitis, Case report

## Abstract

**Background:**

Hashimoto’s encephalitis (HE) is a rare neurological complication of Hashimoto’s thyroiditis (HT), while limbic encephalitis (LE) is an autoimmune inflammatory disorder frequently associated with anti-neuronal antibodies. The glutamate receptor α-amino-3-hydroxy-5-methyl-4-isoxazole-propionic acid receptor (AMPAR) is important for synaptic transmission, memory, and learning. The etiology of HE remains unclear. We present a case of HE with antibodies to AMPAR2 both in the serum and cerebrospinal fluid.

**Case presentation:**

The patient presented with progressive memory loss and subsequently went into a coma. Magnetic resonance imaging revealed temporal lobe and hippocampal lesions, while the electrocardiogram showed paroxysmal delta waves. Elevated serum levels of antibodies against thyroid globulin, thyroid peroxidase, and thyroid stimulating receptor were also noted. Ultrasonography showed enlargement of the thyroid gland. Therefore, the diagnosis was established as HE. Both the CSF and serum samples of the patient tested positive for antibodies to the cell-surface antigen AMPAR2. Intravenous injection of immunoglobulin followed by dexamethasone treatment resulted in recovery from the coma. Follow-up examination three months later showed some improvement of memory. To our knowledge, this is the first report on the detection of AMPAR2 antibodies in HE.

**Conclusions:**

Our findings suggest that antibodies to AMPAR2 may be involved in the pathogenesis of HE. Elevated levels of thyroid antibodies possibly cause immune dysfunction, leading to the production of anti-AMPAR2 antibodies that are detrimental to the neurons. We believe that encephalitis patients with thyroid abnormalities should undergo screening for anti-neuronal antibodies, and early immune therapy may improve prognosis.

## Background

Hashimoto’s thyroiditis (HT) is the most common type of autoimmune thyroid disease (AITD); it is characterized by the presence of high titers of anti-thyroid antibodies in the blood [1]. The neurological complication of HT, namely, Hashimoto’s encephalitis (HE,) was first reported in the 1960’s by Brain et al. [2]. The clinical presentations of HE are diverse, ranging from focal signs similar to those manifested in stroke-like events to those reflecting diffuse panencephalitis, such as altered cognition and psychosis [3, 4].

Limbic encephalitis (LE) is an autoimmune inflammatory disorder of the limbic system, involving the medial temporal lobe, amygdala, and cingulate gyri. Clinically, it is clinically manifested by short-term memory deficit, psychosis, and seizures [5]. Recent studies have suggested that the pathogenesis of LE is mediated by anti-neuronal antibodies, including antibodies to both intra-neuronal and cell-surface antigens. Intra-neuronal antigens are usually paraneoplastic, while cell-surface antigens are thought to be immune-mediated [5–7]. The glutamate receptor α-amino-3-hydroxy-5-methyl-4-isoxazole-propionic acid receptor (AMPAR) is a cell-surface ionotropic receptor that plays important roles in synaptic transmission, memory, and learning [8]. Anti-AMPAR encephalitis was first reported in 2009 in a cohort study of ten patients [9]. Exposure of neurons to the antibodies causes a significant decrease in the total amount of AMPAR cluster and synaptic locations of GluA1- and GluA2-containing AMPARs [10], which demonstrates the pathogenic effect of anti-AMPAR antibodies.

Even to date, the pathogenesis of HE is largely unclear and widely debated. Several mechanisms, such as vasculitis, cerebral hypoperfusion and cerebral tissue specific autoimmunity, have been postulated thus far. In this paper, we present a case of HE with evidence of antibodies against AMPAR2 positive both in the serum and cerebrospinal fluid (CSF) [4].

## Case presentation

A 54-year-old previously healthy woman was admitted to our hospital for progressive cognitive decline and memory loss since 5 days. The patient’s symptoms started with the inability to remember the names and the functions of the condiments on the first day. The next day, the patient had difficulty in recollecting the names of her close relatives, in addition to dizziness and fatigue. At the time of admission, the patient was conscious, but confused. History taking revealed no fever, headache, or significant weight loss during the past three months, and no family history of auto-immune diseases. On further clinical examination, long-term and short-term memory, the ability of calculation, as well as temporal and spatial perception were found to be impaired. No other neurological signs were present, expect for a positive Chaddok sign on the left side.

**Fig. 1:**
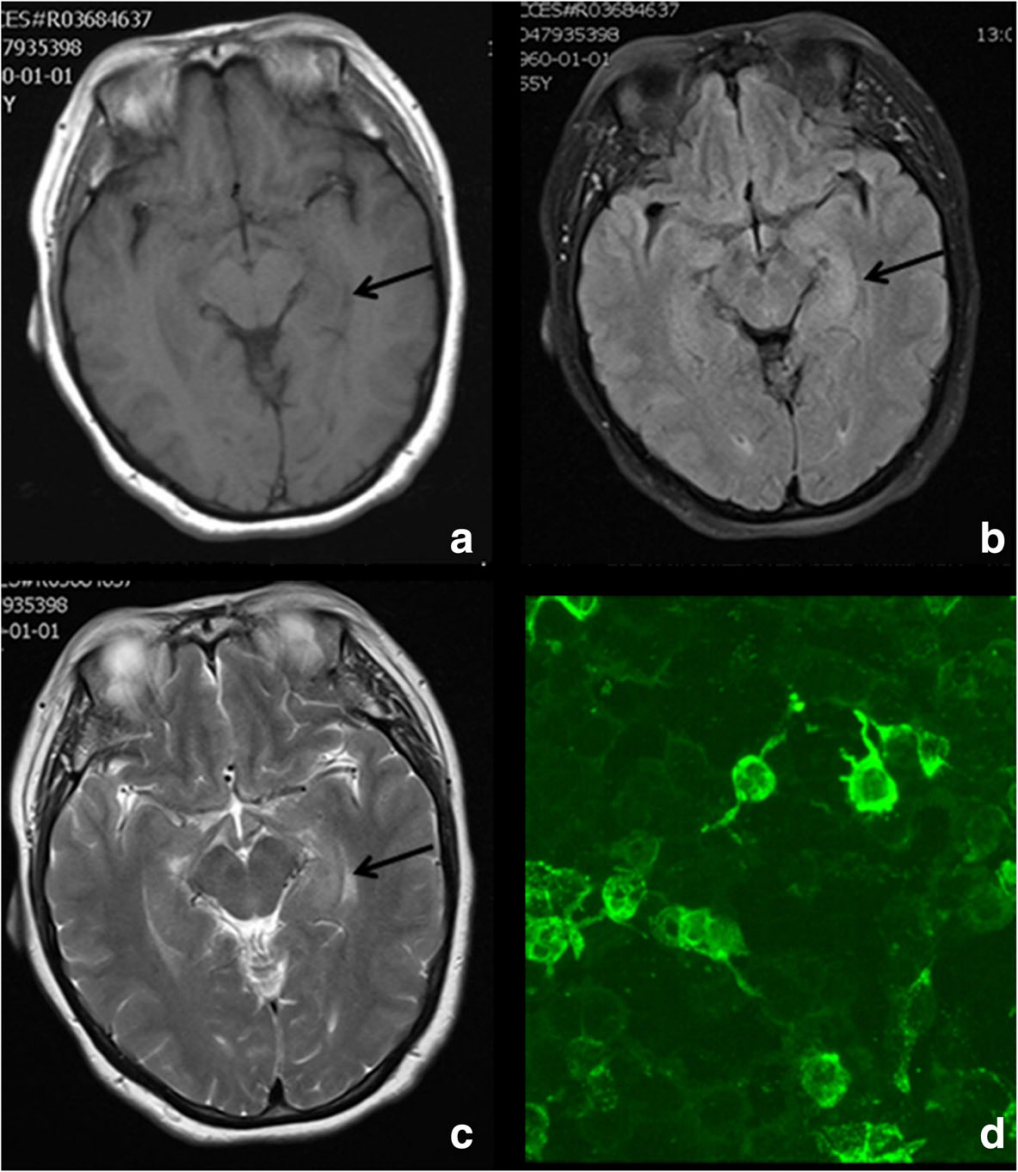
**a**-**d**. Axonal magnetic resonance imaging (MRI) scans revealed patchy lesions in the left temporal lobe and hippocampus. The lesions were represented by areas of low signal intensity on T1-weighted imaging (**a**) and high signal intensity on T2-weighted imaging (**b**) sequence and high signal intensity on the fluid-attenuated inversion recovery (FLAIR) sequence (**c**). Anti-AMPAR2 antibodies in the serum of the patient was positive, as tested by CBA method (**d**)

The first magnetic resonance image (MRI) obtained at the local hospital showed normal findings. The second MRI repeated on the next day at our hospital revealed patchy lesions in the left temporal lobe and hippocampal area. The lesions showed high signal intensity on T1- and T2-weighted imaging and high signal intensity on the fluid-attenuated inversion recovery (FLAIR) sequence (Fig 1a-c). Electrocardiography (EEG) revealed paroxysmal delta waves in the left temporal lobe. Ultrasonographic examination showed enlargement of the thyroid gland (left thyroid lobe: 16 mm × 6 mm, and right thyroid lobe: 15 mm × 16 mm × 43 mm), with irregular echogenicity. However, ultrasonography examination of the abdominal organs, including the liver, kidney, pancreas and uterus, as well as other organs, such as mammary glands, did not reveal any abnormality. Pulmonary computed tomography (CT) scan was negative for neoplasia.

The findings of routine blood tests were normal, except for slightly low levels of sodium (133.9, normal range: 137–147 mmol/L) and chloride (97, normal range: 99–110 mmol/L). The screening assay for viral infection showed elevated levels of antibodies against herpes simplex virus I (HSV I) (8.023 S/CO, normal range: <1.00 S/CO), rubella virus (0.927 S/CO, normal range: <1.00 S/CO), and cytomegalovirus (CMV) (6.332 S/CO, normal range, normal range: <1.00 S/CO) in the serum but not in the CSF. The results of the lumbar puncture test showed increased intracranial pressure (230 mm H_2_O; normal range: 70–180 mm H_2_O). Routine analysis of the CSF showed increased concentration of protein (0.50 g/L, normal range 0.15–0.45 g/L) and elevated white blood cell count (63 × 10^6^/L, normal range: 0–8 × 10^6^/L), with 89% lymphocytes, 7% eosinophils, and 4% monocytes. The CSF was negative for oligoclonal bands. Further, the levels of the immunoglobulin (Ig) G were also found to be slightly increased (49.1 mg/L; normal range: 0–34.0 mg/L) in the CSF. The results of the autoimmune encephalitis test using cell-based assay (CBA) for antibodies against AMPAR2 were positive for both the serum and CSF (Fig 1d), but negative for other antibodies, including NMDA-R, CASPR2, AMPA1-R, and LGI1. Screening tests were also negative for paraneoplastic markers, including Hu, Yo, Ri, paraneoplastic Ma2 (PNMA2), CV2/collapsing response mediator 5 (CRMP5), and amphiphysin. Additionally, tests for tuberculosis were negative. Thyroid function-related tests showed slightly lower levels of thyroid stimulating hormones (TSH) (0.26 mIU/L; normal range: 0.27–4.2 mIU/L), while the serum levels of free T3 and T4 were within the normal range. Levels of the antibodies against the thyroid globulin (TG) (2006 IU/ml, normal range <115 IU/ml), thyroid peroxidase (TPO) (276.9 IU/ml, normal range <35 IU/ml), and thyroid stimulating receptor (TSHR) (13.6 IU/ml, normal range 0.3-1.75 IU/ml) were found to be elevated. Tests for antibodies against and antigen of hepatitis B and C virus were all negative. Antibody tests for human immunodeficiency virus (HIV) and syphilis were also negative, as were the screening tests for the tumor biomarkers. The results of routine urine analysis and tests for liver and kidney function were normal. The blood levels of lipids and glucose were also within the normal range. The erythrocyte sedimentation rate and levels of the C-reactive proteins were also within the normal range.

On the basis of the results of the preliminary physical examination and the laboratory tests conducted on the day of admission, viral encephalitis was suspected. Accordingly, the patient was treated with the anti-virus drug acyclovir. However, there was no significant relief in the patient’s symptoms. Four days later, the patient’s conditions worsened and the patient went into a coma; therefore, she was shifted to the intensive care unit. There, anti-AMPAR2 antibodies were detected in the serum and CSF, and accordingly, the patient was treated with intravenous injection of immunoglobulin (IVIG) for 3 days followed by dexamethasone. Once the IVIG treatment was initiated, the patient recovered from coma. Follow-up examination after 3 months showed that her memory function had improved to some extent, but not completely restored.

## Discussion

In this paper, we presented a case of HE in which both serum and CSF were positive for antibodies to AMPAR2. HE is a rare neurological complication of HT, and approximately, one-third of HE cases occur in the euthyroid state of HT [11]. Although HE has been a known clinical entity since 50 years, its etiology is still not fully understood.

Our patient had progressive memory loss and eventually slipped into a coma. MRI scan showed lesions in the temporal lobe and hippocampus, while the EEG showed paroxysmal delta waves. Furthermore, the patient had elevated serum levels of antibodies to TG, TPO, and TSHR. In the light of these findings, the diagnosis was established as HE. High levels of anti-thyroid antibodies have been previously reported in the CSF of HE patients [12].

Moreover, anti-thyroid antibodies have been showed to bind to astrocytes [13], suggesting a direct pathogenic link between the two. However, the levels of anti-thyroid antibodies do not correlate with the severity of the clinical syndrome, and immunotherapy does not always reduce the levels of anti-thyroid antibodies despite reliving the clinical symptoms [14]; these findings suggest that there are more mechanisms, hitherto undiscovered, may be involved in the pathogenesis of HE. It has been suggested that anti-thyroid antibodies could serve as markers for autoimmune disease in the brain and anti-neuronal auto-antibodies may exist in HE. Oide et al. showed that serum of HE patients contained an anti-neuronal autoantibody that reacted with a 36-kDa antigenic protein from human cerebral cortex [15]. Moreover, the CSF of HE patients has been shown to test positive for other anti-neuronal auto-antibodies against enzymes expressed by neurons [16].

The antibodies to the cell surface antigen AMPAR2 were found positive both in the CSF and in the serum of this patient. To our knowledge, this is the first report on the detection of AMPAR2 antibodies in HE patients. Since the first report of AMPAR encephalitis in 2009, no more than 50 cases have been reported thus far [17]. One peculiar feature of this case is that the clinical presentation in this case is also consistent with the diagnosis of AMPAR2 LE. More than half of the cases of AMPAR encephalitis show evidence of coexistent tumor [17, 18]. Therefore, to rule out coexistent neoplasia, we performed ultrasonography of the abdominal organs and mammary glands in addition to pulmonary CT. However, no evidence of tumors was obtained in these scans. Moreover, screening for tumor markers and paraneoplastic auto-antibodies also did not yield any significant findings. Thus, caner was ruled out in this patient. In the light of our experience in this case, we speculate that the elevated levels of anti-thyroid antibodies may have caused immune dysfunction in the brain; this may have triggered the production of AMPAR2 antibodies, which, in turn, have a detrimental effect on neurons. It would be worthwhile to further investigate whether anti-thyroid antibodies have a causative relationship with the\production of AMPAR2 antibodies.

Vasculitis is another leading theory used to explain the etiology of HE. An autopsy study in a 77-year-old female patient showed infiltration of inflammatory CD3-positive T-lymphocytes in the brain stem, cerebellum, and cortex. In line with this hypothesis, increased concentrations of protein were found in the CSF of our patient, indicating the disruption of the blood brain barrier (BBB). Furthermore, increases were noted in the number of monocytes and eosinophils in our patient. Increase of the inflammatory cells in the CSF can produce inflammatory cytokines, which can cause damage to the neurons.

The patient’s symptoms improved after initiation of IVIG treatment. Immunotherapy should be considered as the first-line-treatment for both HE and LE, and most of the patients show immediate response to the treatment, with reversal of the symptoms as well as MRI changes. However, the persistence of the neurologic deficits and eventual fatality may occur depending on the severity of the disease and complication of other organs. This patient’s memory deficits did not completely recover despite administration of immunotherapy. Therefore, IVIG or glucocorticoid treatment alone or their combination is recommended as soon as the diagnosis is established.

## Conclusions

Our findings in this case support the role of inflammatory autoimmunity in the pathogenesis of HE. Anti-thyroid antibodies may cause dysregulation of the immune cells in the brain, leading to the production of antibodies to AMPAR2, which in turn are detrimental to neurons. Our experience in this case also indicates that encephalitis patients with autoimmune thyroid disease should undergo screening for anti-neuronal antibodies and receive early immune therapy for a better prognosis.

## Abbreviations

AMPAR: α-amino-3-hydroxy-5-methyl-4-isoxazole-propionic acid receptor
BBB: Blood–brain barrier
CMV: Cytomegalovirus
CSF: Cerebral spinal fluid
ESR: Erythrocyte sedimentation rate
HE: Hashimoto’s encephalitis
HSV: Herpes simplex virus
HT: Hashimoto’s thyroiditis
Ig: Immunoglobulin
IVIG: Intravenous injection of immunoglobulin
LE: Limbic encephalitis
MRI: Magnetic resonance imaging
TSH: Thyroid stimulating hormones
